# Reaching women in Egypt: a success story

**Published:** 2009-06

**Authors:** Ahmed Mousa, Gamal Ezz El Arab, Ebtehal Rashad

**Affiliations:** Lecturer, Department of Ophthalmology, King Saud University, PO Box 245, 11411, Riyadh, Kingdom of Saudi Arabia. Email: ahmousa7@gmail.com; Medical Director, Al Noor Magrabi Foundation, Cairo, Egypt.; Medical Anthropology Consultant, Al Noor Magrabi Foundation, Cairo, Egypt.

**Figure:**
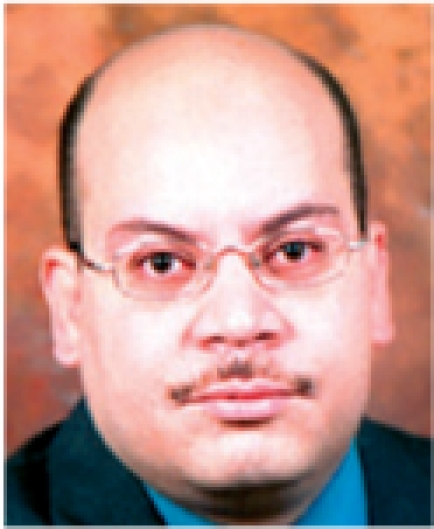


**Figure:**
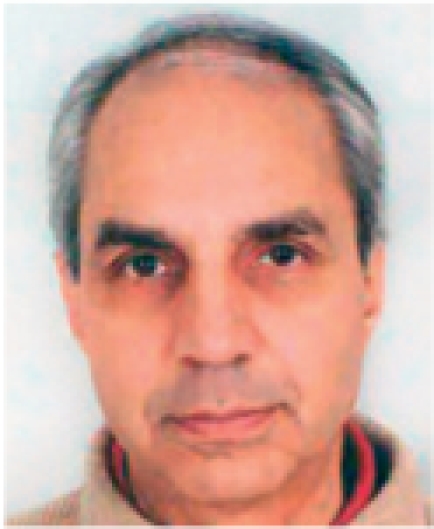


**Figure:**
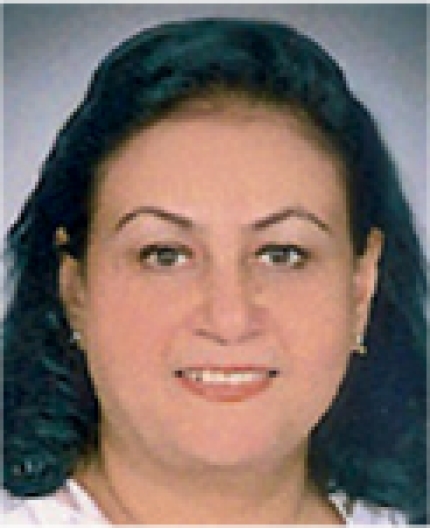


Women in Egypt are more likely than men to suffer from low vision or blindness from avoidable causes.^1^–^3^ This is, in large part, because women are not using eye care services as frequently as men, especially in rural areas.^4^–^5^ A 2002 community-based survey of 4,500 people in Al Minya Governorate, Upper Egypt showed that the prevalence of cataract in women was double that in men and that trachomatous trichiasis was four times as prevalent in women as in men.^3^

Egypt has a large number of eye care providers, even in rural and suburban areas, but a very low uptake of eye care services. On the provider side, this is due to poor clinical outcomes and poor interaction with patients, both of which contribute to fear of surgery. On the patient side, low uptake is due to lack of family support, stigmatisation, and the fact that older persons (who are more likely to be visually impaired or blind) are less able to influence decisions within households. Many also believe that they are too old for surgery. As a result, few patients actually seek eye care.

From formative research conducted in Al Minya, we know that women face particular difficulties in accessing services:

- They are less aware of services, in part due to higher illiteracy rates.
- They have more limited access than men to the family's financial resources.
- They have greater fear of surgery.
- They have more responsibilities towards their home and children, making it difficult for them to leave home.

**Figure:**
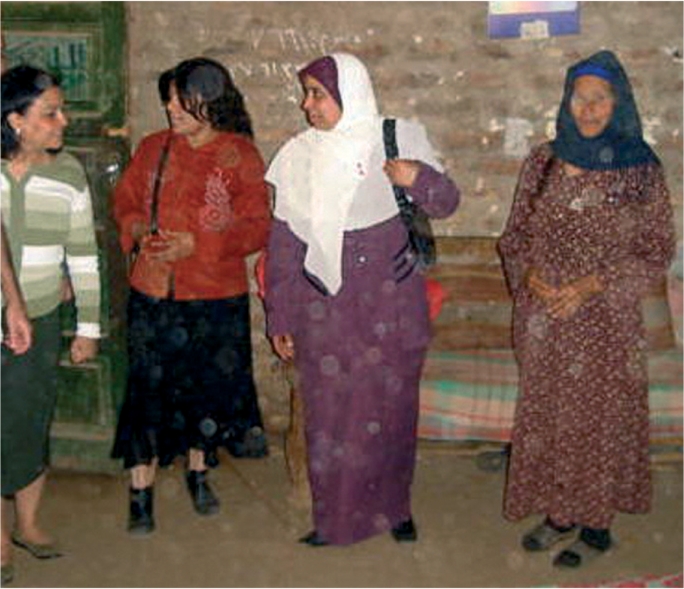
Focusing on women within households. EGYPT

Mahmoud Abo Al Saud

**Figure:**
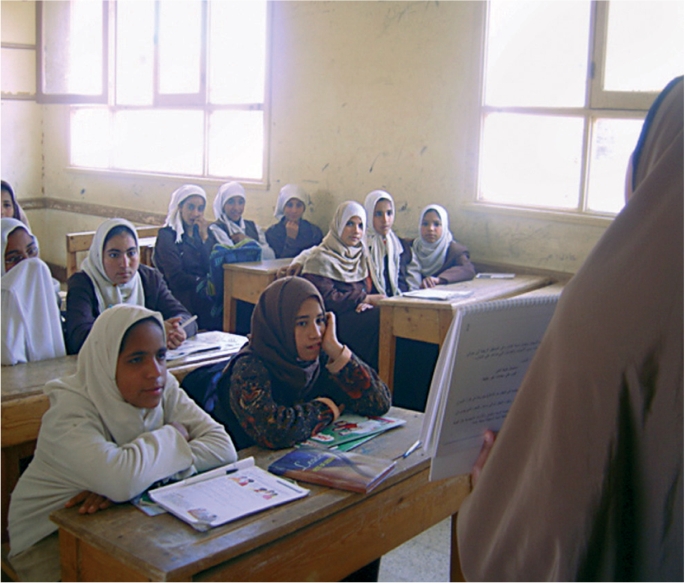
Talking to female students in one of the intervention villages. EGYPT

Mahmoud Abo Al Saud

**Table 1:** Al Minya community survey 2002: prevalence of cataract and trichiasis

No. of people presenting	Men (%)	Women (%)
Cataract	142 (29.1)	346 (70.9)
Trachomatous trichiasis	37 (20.0)	148 (80.0)
**total**	**179 (26.6)**	**494 (73.4)**

## Addressing the problem

Based on the results of the 2002 community survey in Al Minya and the formative research later conducted, a multidisciplinary team from Al Noor Magrabi Foundation in Egypt was formed to investigate the effectiveness of an integrated programme to improve the eye health of women in the region. Funding was provided by the Canadian Institute for Health Research and Al Noor Magrabi Foundation; international consultants from the University of British Columbia and the Kilimanjaro Centre for Community Ophthalmology provided support.

The team identified two intervention villages (population 12,000 each) in the Samalout district in Al Minya governorate and two similar villages in the same district to act as controls. At the time of the intervention (2006–7), the available eye care services consisted of primary health care units in each village, staffed by a general medical practitioner who could refer eye patients to Samalout Eye Hospital or Al Minya Eye Hospital. Both hospitals conducted cataract and trichiasis operations; however, only two cataract operations were conducted per day and only Snellen trichiasis surgery was possible (a technique known to have a high rate of recurrence).

The intervention had two major components: using women to reach women in the community and strengthening the local eye care system.

### 1  Using women to reach women in the community

As a first step, the team established a good relationship, through various meetings and presentations, with local policy makers, local health authorities, community leaders, local non-government organisations (NGOs), and local health and eye care providers. This enabled us to work directly with communities to identify the best people to assist with the intervention.

The team chose to use women to reach female community members in the intervention villages, as they would be able to enter homes and meet with women without coming into conflict with regional customs. Candidates with previous experience in community development projects were selected and trained by the authors over a period of three days.

A total of 42 women were trained, of whom 30 were finally selected. The women, known as health visitors, visited a total of 2,354 households (90 per cent of the population in the two intervention villages) from March to December 2007.

During each visit, health visitors explained to women that saving or restoring their own sight would benefit the whole family. They also talked to husbands, fathers, and sons about the importance of seeking eye care for the women in their household. Each family received a variety of educational materials, including a calendar with illustrations relating to eye care.

**Figure:**
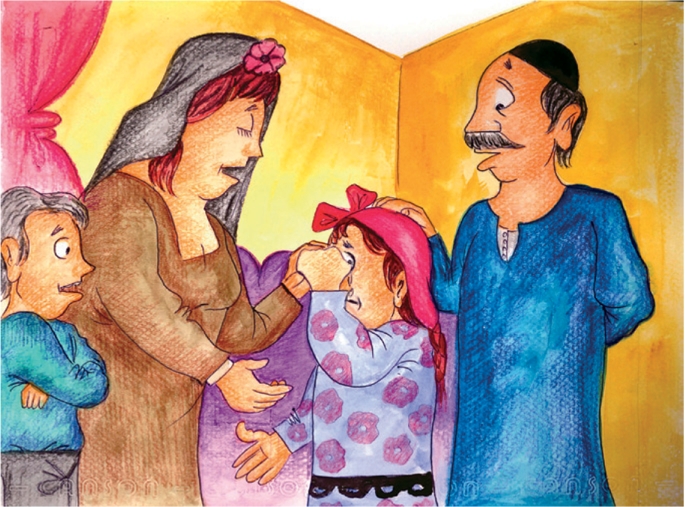
One of the illustrations used in the health education materials given to families. This formed part of a story about a family with trachoma. EGYPT

Editor: Mahmoud Mansour

**Figure:**
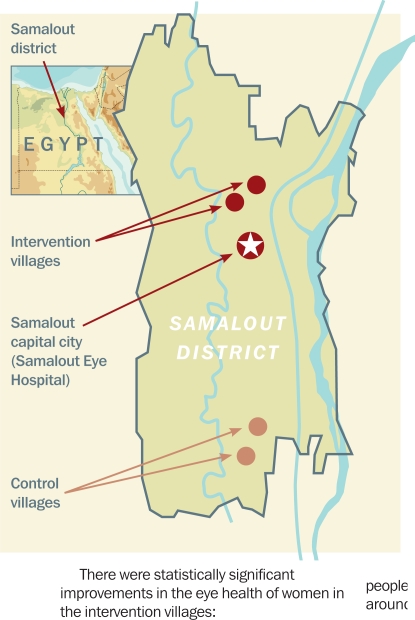
Figure 1. Location of intervention and control villages in Samalout district

During the same visit, health visitors assessed vision using E charts and looked for signs of cataract and trichiasis. They recorded the names of people who needed further care and referred them to the local hospital (Samalout Eye Hospital).

In total, the health visitors identified 563 people who needed eye care services: 302 with cataract, 97 with trichiasis, and 164 with other eye conditions. Overall, 72 per cent of those who needed eye care services were women.

Community-based organisations helped with transport to hospital by providing financial and logistical support. In many instances, the health visitors themselves, thanks to their knowledge of the local community, were able to arrange transport and accompany community members on the day of their appointment.

### 2  Strengthening the local eye care system

The nearest hospital, Salmalout Eye Hospital, needed additional equipment and instruments as well as more consumables in order to cope with the expected increase in demand. The community also lacked trust in the hospital, which meant that the team needed to provide training and support to improve the quality of services.

The team donated five cataract sets and many other surgical instruments to the hospital. Of the four ophthalmologists there, two received training at Magrabi Hospital in Cairo to upgrade their skills. The training included improved techniques in diagnosis and small incision cataract surgery. Separately, all eye department staff members were trained to manage patients and communicate with them better in order to improve patients' experience at the hospital. Staff members were also informed about the project and about the need for more women to receive eye care.

Experienced ophthalmologists from Magrabi Hospital, Cairo assisted and supervised the first 132 operations at Samalout Eye Hospital; this further helped to build the skills and confidence of local surgeons.

In addition, a system was developed to increase the number of outpatient visits per day and the number of operations that could be performed in one day.

## Impact

To measure the reduction in blindness of both men and women, we conducted surveys before and after the intervention in both the intervention and control villages. A calculated sample of 269 adults in the intervention villages and 269 in the control villages were surveyed.

There were statistically significant improvements in the eye health of women in the intervention villages:

- The prevalence of blindness (visual acuity [VA] <3/60) had decreased by 9.0 per cent (p = 0.006): from 12.2 to 3.2 per cent.
- The prevalence of visual impairment (VA <6/18 to 3/60) had decreased by 14.1 per cent (p = 0.01): from 45.5 to 31.4 per cent.
- The prevalence of cataract had decreased by 18.4 per cent (p = 0.0003): from 35.6 to 17.2 per cent.
- The prevalence of trachomatous trichiasis had decreasedby 8.2 per cent (p = 0.0012): from 11.9 to 3.7 per cent.

The eye health of men in the intervention villages had also improved, with statistically significant decreases in both visual impairment and cataract:

- The prevalence of visual impairment (VA <6/18 to 3/60) had decreased by 13.7 per cent (p = 0.0024): from 32.7 to 19.0 per cent.
- The prevalence of cataract had decreased by 13.2 per cent (p = 0.021): from 23.6 to 10.4 per cent.

There were no statistically significant changes in the prevalence of blindness, visual impairment, cataract, or trachomatous trichiasis among men or women in the control villages.

### Increased awareness

Between November 2006 and mid-2008, awareness of blinding diseases and gender-sensitive approaches had significantly increased at both community and political level. With increasing awareness, more volunteers and community leaders (including two local NGOs) became actively involved in eye health education activities. Government leaders, including the governor of Al Minya and Egypt's deputy minister of health, became more dedicated to combating blindness in the district. They encouraged relocation of ophthalmologists to rural areas, developed mechanisms for early detection and referral of cases from primary health care units, and assessed hospital needs in terms of equipment, training, and facilities.

### Improved capacity

As a result of the interventions at Samalout Eye Hospital, more outpatients were seen and a higher volume of operations with good outcomes were performed in 2008 than in 2006, before the intervention.

The average number of outpatients per day increased from 275 to 425, while the number of cataract and trichiasis operations per year increased from 289 to 896. Of the cataract operations performed on people from the intervention villages, around 67 per cent were on women.

## Conclusion

Although this intervention focused on women, men also benefited. In addition, we have learned that people should be supported in seeking services, for example by helping them with transport. Health systems should also be strengthened to absorb the increased demand for services; otherwise, communities may get more frustrated and mistrust eye care providers. We believe our project was successful because it combined health education, capacity building of local providers, and breaking down of barriers in a single, integrated programme.
